# Epidemiology of body dysmorphic disorder among adolescents: A study of their cognitive functions

**DOI:** 10.1002/brb3.1710

**Published:** 2022-03-20

**Authors:** Soran Rajabi, Leila Kamran, Mahnaz Joukar KamalAbadi

**Affiliations:** ^1^ General Psychology Persian Gulf University Bushehr Iran; ^2^ Persian Gulf University Bushehr Iran

**Keywords:** attention, body dysmorphic disorder, executive functions, problem‐solving, Wisconsin

## Abstract

**Background:**

Body dysmorphic disorder (BDD) is characterized by a preoccupation with an imagined defect in one's appearance. In case of a slight physical anomaly, the person would experience an excessive concern. This disorder causes cognitive dysfunction.

**Purpose:**

The aim of this study was to examine epidemiology of body dysmorphic disorder among students at secondary schools of the first and second stage in Shiraz, Iran. It also compares executive functions in students with BDD to healthy students.

**Methods:**

The Body Dysmorphic Disorder Questionnaire (BDDQ), Stroop Color and Word Test (SCWT),Wisconsin Card Sorting Test (WCST), Tower of London test (ToL), and Trail Making Test (TMT) were measured in participants with BDD (*N* = 52; *M*_age_ = 16.20; *SD* = 1.03) and healthy control group (*N* = 52; *M*_age_ = 15.91; *SD* = 0.96).

**Results:**

The frequency of BDD was significantly higher in women than men (14.8% vs. 6.8%), and its prevalence was 10.4% in total. There was a significant difference between the two groups of students concerning attentional set‐shifting, inhibition of cognitive interference, visual‐spatial searching, and sequencing, but not problem‐solving tasks.

**Conclusions:**

Students with BDD have cognitive deficits, which need to be addressed in cognitive rehabilitation.

## Graphical abstract

Body dysmorphic disorder is characterized by a preoccupation with an imagined defect in one's appearance. In case of a slight physical anomaly, the person would experience an excessive concern. This disorder causes cognitive dysfunction. The present study is an attempt to examine epidemiology of body dysmorphic disorder among students at secondary schools of the first and second stage in Shiraz, Iran, and it also compares executive functions in students with body dysmorphic disorder to healthy students. The results of multivariate analysis of variance show that there is a significant difference between the two groups of students concerning set‐shifting, inhibition, visual‐spatial searching (attention), but not problem‐solving task. It can be concluded that students with BDD have cognitive deficits in brain, which needs to be addressed in clinical work.

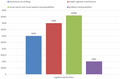

## 1 INTRODUCTION

All through history, beautiful people have usually gained much more respect. In spite of the fact that the definition of an ideal body shape has changed over time, the value of bodily appearance attraction has remained unvarying. Therefore, everyone usually expresses a degree of concern about physical appearance (Toh, Rossell, & Castle, 2009) and wishes for changes in parts of their body. Such tendencies have turned into an obsessive behavior (Tomas‐Aragones & Marron, 2016), which is called body dysmorphic disorders (BDD) in psychiatry and psychopathology textbooks. This mental disorder causes individuals to describe their bodily appearance often as unattractive and ugly. They are obsessed with one or more aspects of their bodily appearance acknowledging their own deformities (Labuschagne, Castle, & Rossell, 2011). Extreme degrees of repetitive behaviors (e.g., constant self‐examination of one's bodily appearance) and mental acts (e.g., comparing oneself with others) are regarded as symptoms of BDD (Abramowitz & Jacoby, 2015).

Having an incompatible self‐image or a “non‐articulate view” o

f self‐makes it difficult for individuals to be secure about themselves. Thus, they have a difficulty synthesizing self‐views into a coherent picture of self, leading to overcompensations in decisions about self‐worth, as individuals tend to engage in all‐or‐nothing thinking (Labuschagne et al., 2011).

The current literature is lacking epidemiology of BDD in patients since it is thought that BDD as a mental disorder occurs in very rare situations (Jefferies‐Sewell, Chamberlain, Fineberg, & Laws, 2017) which is not confirmed by epidemiological data. The prevalence rate of BDD has been reported between 1.7% and 2.4% (Kelly et al., 2017). Moreover, the comparison of men and women in terms of the severity of the BDD symptoms as well as the extent of comorbid disorders showed female patients reported more generalized anxiety disorder (GAD) symptoms (Schneider, Mond, Turner, & Hudson, 2019).

The literature suggests that BDD and obsessive–compulsive disorder (OCD) share common characteristics in terms of clinical and familial histories as well as response to treatments. However, recent studies on the classification of BDD symptoms in adolescents have suggested that symptoms of BDD appear to form a separate factor that is correlated with symptoms of affective disorders and eating disorders (Schneider, Baillie, Mond, Turner, & Hudson, 2018). The fifth Diagnostic and Statistical Manual of Mental Disorders (DSM‐5) has placed this mental disorder in the novel category of “OCD and related disorders” such as OCD, hoarding disorders, trichotillomania (hair‐pulling disorders), excoriation disorders (also referred to as chronic skin picking or dermatillomania), and numerous known or unknown diagnostic cases (Schneider et al., 2018). BDD typically begins during adolescence with damaging effects on education, emotions, as well as social functions. In teen years, BDD is distinguished by more severe symptoms such as increased comorbid disorders during natural life and high rates of suicide attempts than adulthood (Krebs et al., 2017).

To the best of our knowledge, there are not enough studies in literature investigating BDD etiology as well as its underlying causes. Conversely, cognitive models have reflected the potential role of certain cognitive impairments in BDD development and maintenance (Greenberg et al., 2018; Jefferies‐Sewell et al., 2017). In functional neuroimaging studies, OCD symptoms are suggestive of increased activity in orbitofrontal cortex, caudate nucleus, thalamus, and anterior cingulate gyrus (Saxena, Brody, Schwartz, & Baxter, 1998).

Executive functions are usually defined as a set of high‐level abilities associated with implementing, monitoring, and regulating targeted behaviors (Lopez et al., 2008). In particular, functions such as organizing, decision‐making, active memory, motor‐control preservation and conversion, time perception, future predictions, reconstruction, internal language, and problem‐solving can be regarded the most important neuropsychological‐executive functions that can help individuals to use learning practices and action intelligence (Barkley, 1998). Most studies in this domain were conducted on memory disorders (Osman, Cooper, Hackmann, & Veale, 2004), social information processing (Buhlmann, McNally, Wilhelm, & Florin, 2002), perceived physical excitement (Buhlmann, McNally, Etcoff, Tuschen‐Caffier, & Wilhelm, 2004), self‐discrepancy (Veale, Haro, & Lambrou, 2003), selective controlled processing and self‐centered involuntary behaviors (Schmidtke, Schorb, Winkelmann, & Hohagen, 1998), verbal and nonverbal learning and memory (Deckersbach et al., 2000), set‐shifting (Kerwin, Hovav, Hellemann, & Feusner, 2014), and metacognition (Osman et al., 2004).

Tower of London (ToL) test (planning ability), Stroop Color, and Word Test (SCWT) (avoidance response) showed that individuals suffering from OCD and BDD reported problems in assessing executive functions (Schneider et al., 2018). However, the presence of compulsive double‐checking and obsessive thoughts of accuracy is likely to help groups with OCD make fewer errors in some cognitive tests (Soyata, Akisik, Inhanli, Noyan, & Ucok, 2018). Jefferies‐Sewell et al. (2017) found cognitive deficits in people with BDD in terms of set‐shifting, avoidance, reaction time, executive planning, and an effective desire for a stimulus with a negative capacity. Kashyap, Kumar, Kandavel, and Reddy (2013) also found that individuals with OCD had deficits in visuospatial short‐term memory, planning time, conceptualization, conflict resolution/response inhibition, decision‐making, and encrypting nonverbal memory caused mainly by a functional disorder. They consequently would encounter problems in planning, designing strategies, and organizing stimuli as well as regulating cognitive resources to meet maximum productivity. They found that patients with BDD had impairments in spatial working memory and thinking speed while having access to short‐term memory capacity, thinking speed, and visual memory similar to the control group. Assessing spatial planning activity similarly showed deficits in planning ability in individuals with BDD (Dunai, Labuschagne, Castle, Kyrios, & Rossell, 2010).

Body dysmorphic disorders needs further support (Sadock, Sadock, & Ruiz, 2015), and research to date has been poor in terms of sample size and has not examined cognitive functions among adolescents; there has also been no research on the epidemiology of BDD and executive functions in adolescents in Iran. More research is needed to determine the cognitive functions among individuals with BDD. The present study aimed to measure cognitive functions in adolescents with BDD by means of cognitive tests such as WCST and TMT. More specifically, the present study was to determine and compare the attentional set‐shifting, ability to inhibit cognitive interference, visual search and visual‐spatial sequencing or scanning abilities, and the problem‐solving abilities of two groups of adolescents, those with BDD diagnosis and those without any body dysmorphic symptoms.

## 2 METHODS

### 2.1 Participants

Five hundred male (*n* = 250) and female (*n* = 250) students were recruited via multi‐stage cluster sampling. They were studying at secondary schools of the first and second stage in Shiraz, Iran. After diagnosing students with BDD, 52 students (37 females and 15 males) were assigned to BDD group and 52 healthy students (37 females and 15 males) were assigned to the healthy control group (HC). The two groups were homogenous in terms of age and gender.

The diagnosis of BDD was based on the body dysmorphic disorder questionnaire and DSM‐5 criteria considering differential diagnostic criteria. To assess mental disorders, the second author administered the structured clinical interview for DSM‐5 disorders, clinical trials version (SCID‐5‐CT) (First, Williams, Karg, & Spitzer, 2015). The exclusion criteria comprised those who (a) used medication under a condition, (b) had a comorbid psychotic disorders or substance use disorders (due to comorbidity with distinct neurological disorders (Sadock et al., 2015), (c) had a neurological disorder and/or visual problems. All the required institutional review board approved consent forms, which were signed by the participants, parents, and teachers.

### 2.2 Test materials

#### 2.2.1 The Body Dysmorphic Disorder Questionnaire (BDDQ)

The Body Dysmorphic Disorder Questionnaire is a brief self‐report measure (five questions), which is derived from the DSM‐IV diagnostic criteria. Close‐ended questions investigate the extent to which the respondents' appearance is a source of their obsession and, if so, they measure the degree to which it causes distress or interference with their social or occupational functioning (Phillips, 2009). A BDDQ score of 4 is equal to the fulfillment of the BDD criteria and thus considered a positive BDD screening (Brohede, Wingren, Wijma, & Wijma, 2013). The questionnaire has been validated by several psychiatric research. For example, it was validated in a psychiatric outpatient sample (*n* = 66), displaying high sensitivity (100%) and specificity (89%) (Phillips, 1995). In a psychiatric inpatient sample (*n* = 122), the sensitivity was 100% and the specificity was 93% (Grant, Kim, & Crow, 2001). A slightly modified version of the questionnaire was validated in a dermatology patient sample (*n* = 46) with high sensitivity and specificity (100% and 92%, respectively) (Dufresne, Phillips, Vittorio, & Wilkel, 2001). The BDDQ has been widely used for BDD screening, for example, with 1,000 dermatology and plastic surgery outpatients (Vulink et al., 2006), 100 psychiatric inpatients (Conroy et al., 2008), 160 patients with maxillofacial problems (Vulink et al., 2008), and 300 dermatological patients (Conrado et al., 2010).

#### 2.2.2 Stroop Color and Word Test (SCWT)

The Stroop Color and Word Test is a common neuropsychological test extensively used for both experimental and clinical purposes (Roy et al., 2018). It assesses executive functions and more specifically the ability to inhibit cognitive interference, which occurs when the processing of a stimulus feature affects the simultaneous processing of another attribute of the same stimulus (Stroop, 1935 Quoted from Scarpina & Tagini, 2017). In particular, the Stroop Test evaluates the ability to suppress habitual responses. In the Stroop Test, color words printed in a different color have to be read aloud; the color of the written words has to be inhibited (e.g., the word “red” printed in green) (Scarpina & Tagini, 2017). Wang, Zhou, Huang, and Yang (2018) estimated the reliability of the alternate form of the Chinese version of SCWT under a congruous condition as well as an incongruous condition (0.91, and 0.91, respectively).

#### 2.2.3 Wisconsin Card Sorting Test (WCST)

The Wisconsin Card Sorting Test (WCST) was designed in 1948 by Grant and Berg as an index of abstract reasoning, concept formation, and response strategies to change contextual contingencies (Eling, Derckx, & Maes, 2008). The participants are asked to sort 64 cards to match either color (red, blue, yellow, or green), form (crosses, circles, triangles, or stars), or number of figures (one, two, three, four). During the task, the sorting rule changes deliberately from color to form or number of figures without informing the participants. The participants have to shift sets accordingly and sort cards following the new sorting rule. Set‐shifting difficulties are indicated by preservative errors; thus, higher scores on this test represent worse performance. The test–retest reliability has been greater than 0.90 in children and adolescents with learning problems over an interval of approximately 2.5 years (Ozonoff, 1995). Lezak, Howieson, Loring, and Fischer (2004) showed that the validity of the test was higher sensitivity than 86% for measuring cognitive impairment after brain damage.

#### 2.2.4 Tower of London test (ToL)

Shallice (1982) as a modification of the Tower of Hanoi designed the Tower of London test. It is primarily used as an executive function task to examine planning ability (Nitschke, Kostering, Finkel, Weiller, & Kaller, 2017). ToL is a 3D apparatus composed of three pegs of different heights and three beads of different colors (red, green, and blue). A specific number of beads can be put on each of the pegs. The subject is asked to move the beads from a starting arrangement of the beads on the pegs, which is always identical, and proceeds to the target arrangement printed on cards presented by an administrator, using a restricted number of moves by subject (Michalec et al., 2017). The internal consistency coefficient (Cronbach's *α*) of the test ranged from 0.33 to 0.60 in the study of Michalec et al. (2017). Kostering, Nitschke, Schumacher, Weiller, and Kaller (2015) assessed test–retest reliability of the ToL in a sample of young, healthy adults over a one‐week interval. For planning accuracy, the Pearson correlation and intraclass correlation coefficients were adequate concerning relative consistency (*r* = .739 and .734, respectively) as the intraclass correlation coefficient only slightly decreased with respect to absolute agreement (*r* = .690).

#### 2.2.5 Trail Making Test (TMT)

Trail Making Test is the most popular neuropsychological test to explore executive functions among older adults (Faria, Alves, & Charchat‐Fichman, 2015). TMT provides information on visual attention, task switching, speed of processing, and mental flexibility (Tombaugh, 2004). It consists of two parts. TMT‐A consists of 25 numbers from 1 to 25 and the subject should draw lines to connect the numbers in an ascending order. The original TMT‐B is composed of 12 numbers and 12 letters. Like Part A, the subject draws lines to connect the circles in an ascending pattern, but with the added task of alternating between the numbers and letters (i.e., 1‐A‐2‐B‐3‐C, etc.). The subject should be instructed to connect the circles as quickly as possible, without lifting the pen or pencil from the paper. Time is recorded as the subject connects the "trail." It is unnecessary to continue the test if he or she has not completed both parts after five minutes. Results for both TMT‐A and ‐B are reported as the number of seconds required to complete the task; therefore, higher scores reveal greater impairment. The validity and reliability of TMT have been evaluated in the healthy older adults (Sánchez‐Cubillo et al., 2009). Wang et al. (2018) showed the reliability of the Chinese version of TMT‐B ranged moderate to excellent with intraclass correlation coefficient of 0.89 and 95% confident interval of 0.63–0.96. In addition, test–retest reliability coefficients for TMT‐A and TMT‐B were estimated as 0.82, and 0.93, respectively.

### 2.3 Statistical analyses

Statistical analyses were performed using the Statistical Package for the Social Sciences (SPSS, version 24) for windows. A Chi‐squared test was used to identify whether frequencies of clinical characteristics and demographics differed significantly between groups. According to the Kolmogorov–Smirnov test, all the examined continuous variables were normally distributed. Therefore, the comparisons between participants' subgroups for variables of cognitive distribution were performed by means of parametric statistical tests. The means of continuous variables between two groups were compared using the multivariate analysis of covariance (MANCOVA). However, prior to analysis of the differences, the data confirmed the homogeneity of the regression slopes, box test, and Levine test as the necessary assumptions for covariance analysis. The insignificant homogeneity assumption of the slopes indicated that the regression slopes were homogeneous. In addition, the approximate parallelism of the regression slopes also confirmed the homogeneity assumption of the regressions and the existence of a linear relationship between the auxiliary random variables (pretest) and the dependent variables (post‐test). The insignificant results of Levin's test also confirmed the equality condition of variances. Moreover, the insignificant results of the box test showed that the homogeneity condition of the variance–covariance matrices has been met.

A *p* < .05 was considered to be statistically significant.

## 3 RESULTS

Table 1 shows the demographic data of grade, age, BDD diagnosis, and BDD subtype which were collected by means of minimization.

**TABLE 1:** Demographic characteristics

Descriptive characteristics	Sample 1		Sample 2		Analysis *T*, *χ* ^2^ *p*‐value (S1, S2)
	Male (*n* = 250)	Female (*n* = 250)	BDD (*n* = 52)	HC (*n* = 52)	
Age (*M* and *SD*)	16.43 (0.83)	16.27 (0.94)	16.09 (1.47)	15.65 (1.22)	.67, .26
Grade (*M* and *SD*)	10.85 (1.06)	10.80 (1.00)	10.75 (0.98)	10.60 (1.18)	.86, .62
BDD (*N* and %)				
HC sample	235 (94.0)	213 (85.2)	–	–	**.001 (*χ* ^2^ = 10.388)**
BDD sample	15 (6.0)	37 (14.8)	–	–	
Comorbidity (*N* and %)				
Mood disorder	–	–	50 (96.1)	2 (3.8)	
Anxiety disorder	–	–	37 (71.1)	1 (1.9)	
OCD	–	–	37(71.1)	0 (33.3)	
Eating disorders	–	–	14 (26.9)	2 (3.8)	
SSD	–	–	15 (28.8)	3 (5.7)	
BDD subtype (*N* and %)				
Skin	33 (22.0)	108 (30.9)	21 (40.4)	10 (19.2)	
Hair	17 (11.3)	24 (6.9)	12 (23.0)	3 (6.9)	
Nose	15 (10.0)	100 (28.6)	18 (34.6)	8 (15.4)	
Mouth	0 (0.0)	6 (1.7)	2 (3.8)	0 (0.0)	
Jaws	2 (1.3)	7 (2.0)	1 (1.9)	0 (0.0)	
Lips	0 (0.0)	11 (3.1)	2 (3.8)	1 (1.9)	
Abdomen	7 (4.7)	37 (10.6)	12 (23.0)	6 (11.5)	
Hip	2 (1.3)	19 (5.4)	9 (17.3)	2 (3.8)	
Breast	0 (0.0)	18 (5.1)	5 (9.6)	1 (1.9)	
Hands	0 (0.0)	16 (4.6)	8 (15.4)	1 (1.9)	
Legs	3 (2.0)	13 (3.7)	7 (13.4)	1 (1.9)	
Penis	8 (5.3)	8 (2.3)	5 (9.6)	2 (3.8)	

Note

*p‐*values and statistic value (*χ* ^2^) for significant finding are reported in bold. S1, S2 = level of significantly for sample 1, level of significantly for sample 2.

Abbreviations: BDD, body dysmorphic disorder; HC, healthy control group; *M*, mean; *n*, sample size; OCD, obsessive–compulsive Disorder; *SD*, standard deviation; SSD, somatic symptom disorder; *T*, *t* test; *χ* ^2^, chi‐square test.

Sociodemographic, epidemiology of BDD, and BDD subtype values of the two samples (sample1 = 500 men and women, and sample2 = 104 people with and without BDD) are reported in Table 1. The two groups (women: *N* = 250; men: *N* = 250) did not significantly differ in terms of age (*p* = .67) and education level (*p* = .86). Also, the two groups (BDD: *n* = 52; non‐BDD: *n* = 52) did not significantly differ in terms of age (*p* = .26) and education level (*p* = .62). The frequency of BDD was significantly higher in women than men (14.8% vs. 6.8%, *χ*2 = 10.388, *p* = .001).

Mood disorders were the highest rates of comorbidity for BDD (96.1). Also, there were the rates of comorbidity of anxiety disorder (71.1), OCD (71.1), eating disorder (26.9), and SSD (28.8) for BDD.

The majority of men (22%, 11.3%, and 10%) were concerned about their skin, hair, and nose, respectively. In women, the skin, nose, and abdomen were the most common concerns (30.9%, 28.6%, and 10.6%), respectively (see Table 1).

Before analyzing the differences, we were assured of the Box test and Levine test as the necessary assumptions for the use of multivariate analysis of variance (MANOVA). Due to the insignificance of Levin's test for all variables, the condition of equality of variances was met. Furthermore, due to the insignificance of the box test for all variables, the condition of homogeneity of variance–covariance matrices was not observed.

In addition, the multivariate analysis of covariance (MANCOVA) of cognitive tasks in the two groups indicated a significant difference at least in one of the variables in WCST (Wilks' Lambda = 0.34, *F*_(4,48)_ = 4.77, *p* < .001), SCWT (Wilks' Lambda = 0.68, *F*_(4,48)_ = 5.79, *p* < .01), TMT (Wilks' Lambda = 0.70, *F*_(4,48)_ = 4.00, *p* < .01), and ToL (Wilks' Lambda = 0.77, *F*_(4,48)_ = 3.19, *p* < .05).

WSCT, SCWT, ToL, and TMT tests scores of the two groups (BDD patients and healthy controls) are reported in Table 2.

**TABLE 2:** Comparisons between BDD patients (*n* = 52) and healthy controls (HC, *n* = 52) for responses to the cognitive tests

Test	Variable	Condition	*M* (*SD*)	*F*	ES	Power
WCST	Number of floor^a^	BDD	2.50 (1.52)	3.70*	0.04	0.95
		HC	2.72 (1.89)			
	Perseveration error	BDD	10.06 (5.49)	6.56**	0.06	0.90
		HC	7.34 (5.11)			
	Incorrect response	BDD	29.24 (8.28)	3.47	0.03	0.88
		HC	25.76 (10.26)			
	Total TIME	BDD	241.96 (101.19)	0.28	0.003	1.00
		HC	231.06 (102.29)			
SCWT	Reaction time	BDD	49.60 (10.07)	7.25**	0.06	0.86
		HC	43.44 (12.64)			
	Error response	BDD	3.26 (7.53)	5.53*	0.05	0.85
		HC	0.72 (1.17)			
	Correct response	BDD	43.32 (8.72)	0.05	0.001	0.84
		HC	43.78 (11.33)			
	Interval score^b^	BDD	2.90 (8.04)	4.03*	0.03	0.91
		HC	0.56 (1.80)			
TMT	Time of part A	BDD	34.72 (12.40)	4.71*	0.05	0.99
		HC	29.48 (11.72)			
	Time of part B^c^	BDD	92.19 (29.66)	4.54*	0.04	0.85
		HC	78.75 (33.25)			
ToL	Time of test	BDD	295.82 (162.77)	4.34*	0.04	0.94
		BDD	249.47 (111.39)			
	Error response	HC	19.43 (8.97)	1.85	0.01	0.87
		BDD	16.86 (9.71)			
	Score^d^	HC	27.84 (3.85)	1.03	0.01	1.00
		BDD	28.67 (4.26)			

Abbreviations: BDD, body dysmorphic disorder; ES, effect size; HC, the healthy control group; *n*, sample size.

^a^ For assesses attentional set‐shifting.

^b^ For assesses the ability to inhibit cognitive interference.

^c^ For assesses the visual search and visual‐spatial sequencing or scanning abilities.

^d^ For assesses the problem‐solving abilities

* *p* < .05.

** *p* < .01.

In WCST test, the number of floor resulted significantly lower in patients than in controls (2.50 ± 1.52 vs. 2.72 ± 1.89, *F* = 3.70, *p* < .04). That is, the BDD patients were significantly weak in attentional set‐shifting. Interval scores of SCWT resulted significantly higher in patients than in controls (2.90 ± 8.04 vs. 0.56 ± 1.80, *F* = 4.03, *p* < .04). That is, the BDD patients were significantly weak in ability to inhibit cognitive interference. In TMT, time of part B resulted significantly higher in patients than in controls (92.19 ± 29.66 vs. 78.75 ± 33.25, *F* = 4.54, *p* < .03). That is, the BDD patients were significantly weak in visual search and visual‐spatial sequencing or scanning abilities. No statistically significant difference was found between the two groups on ToL scores (*p* = .31). In particular, the two groups do not have significant difference in terms of the problem‐solving abilities.

## 4 DISCUSSION AND CONCLUSION

The purpose of this study was to investigate BDD epidemiology among adolescents and determine the relationship between this mental disorder and cognitive functions.

We found that the frequency of BDD was significantly higher in women than men (14.8% vs. 6.8%) and its prevalence was 10.4% in total. The majority of men were concerned about their skin (e.g., facial acne or scarring), hair (e.g., hair loss), and nose (e.g., size and shape). In women, the skin (e.g., facial acne or scarring, wrinkles, color), nose (e.g. size and shape), and abdomen (e.g., abdominal enlargement) were the most common concerns. Our finding is more than 2.4% reported in DSM‐5 (Sadock et al., 2015). Also, the prevalence rate of BDD has been reported between 1.7% and 2.4% in other studies (Kelly et al., 2017). Literature has also reported no differences between men and women in terms of the severity of the BDD symptoms as well as the extent of comorbid disorders (Schneider et al., 2019).

However, BDD needs further support (Sadock et al., 2015) and the current literature is lacking the epidemiology of BDD in patients (Jefferies‐Sewell et al., 2017). Therefore, no conclusion can be safely drawn about its prevalence and the differences between males and females. Perhaps, one of the main reasons why the prevalence rate in our study was higher than other studies is the population sample of adolescents. Studies have shown that BDD is diagnosed by more severe symptoms such as increased comorbid disorders during natural life and high rates of suicide attempts than adulthood (Krebs et al., 2017).

Another result reported in Table 1 was that Mood disorders were the highest rates of comorbidity for BDD. Literature has also reported in both male and female adolescents, BDD is moderately associated with affective disorders, including OCD, anxiety, and depression (Schneider et al., 2018). These researchers conclude that this study highlights the need for future studies examining the classification of BDD to consider developmental and sex differences in their models.

We found that the amounts of WCST sorting (i.e., flexibility) as well as the tendency components, the number of incorrect responses, the total number of attempts, and other errors in individuals with BDD were lower than those do in our healthy individuals. Our findings are similar to those reported by Greenberg et al. (2018). Deficits in set‐shifting in BDD could be explained by specific characteristics of this mental disorder and difficulty in changing a person's attention. Clearly, impaired set‐shifting plays a significant role in the obsessive nature of individuals. A person affected with BDD may fail to abandon one's thoughts associated with bodily appearance and re‐focus on another stimulus. Disrupted set‐shifting in repetitive actions is reflected with the compulsive nature of checking the mirror. Individuals with BDD demonstrated considerable cognitive impairment (attention deficits) which is consistent with the results reported for OCD patients (Jefferies‐Sewell et al., 2017).

One of the main characteristics of OCD is involuntary repetitive behaviors such as obsessive activities, which are related to a disrupted ability in frontal lobes for movement inhibition or cognitive planning. Recent neuroimaging studies have confirmed the hyperactivity of peripheral orbitofrontal cortex pathways, caudate nuclei, and anterolateral cortical cortex in patients with OCD (Saxena et al., 1998). Therefore, it is possible that individuals’ performances in WCST could be affected by peritoneal dorsolateral cortex lesions (Milner, 1963).

In our study, people with BDD had lower mean values in interacting scores compared with normal individuals; thus suggesting that they had lower inhibition rates in responses. Considering SCWT, which indicates abilities in selective attention, alternating attention, and response control, a person, should refrain from giving an automatic response. Therefore, lower scores of individuals with OCD in terms of inhibition ability could be interpreted as their low ability in selective focus, alternating attention, and response control. Such a deficit could be explained by unconscious obsessive thoughts that predominantly engage these individuals in previous and repeated responses and information. The resistance created in their memory also disables them to regain their attention from the previous source, thus disturbing their attention to the correct response. This condition increases the number of errors made during this test. Furthermore, it is likely that OCD individuals consciously sustain their attention to the previous response due to their extreme obsession with the stimuli and risks.

Recent studies have demonstrated that OCD patients face problems using organizing strategies during the encryption of their tacit memories and in situations, encompassing executive functions (see e.g., Abramovitch, Abramowitz, & Mittelman, 2013). These problems have been specifically addressed in assignments (e.g., SCWT) which are generally assumed to stimulate inhibitory processes. In addition, the executive overload model (EOM) for people with OCD suggests that an overflow of obsessive thoughts causes an overloaded executive system, which in turn limits their cognitive capacity and increases the likelihood of obsessive thoughts during the test (Abramowitz & Jacoby, 2015). According to the model of Abramovitch, Dar, Hermesh, and Schweiger (2012), OCD patients fail to make the best use of their cognitive capacity in a complete manner due to the intensity of obsessive thoughts as well as the inception of these thoughts during the test. As a result, their performance was reported to be lower than that of the control group.

Our study demonstrated a significant difference between both groups during the implementation of section B of TMT; thus implying that individuals with BDD had a weaker visuospatial search than those with no disorders. This is supported by several studies (Deckersbach et al., 2000; Greenberg et al., 2018; Kerwin et al., 2014; Soyata et al., 2018).

It is believed that people with BDD focus much more on visual details, while they lack overall visual processing. Such a tendency is usually observed in the phenomenology of individuals with BDD. For example, when they look in the mirror, their attention often focuses immediately on the perceived defects even if they are standing near the mirror but unable to see the larger image of their body shape. Studies have shown that deficits in visuospatial organizing might lead to over‐focused attention in BDD, thus suggesting confirms neurological disorders in BDD patients (Greenberg et al., 2018). In addition, other findings (Schneider et al., 2018) suggest that BDD and OCD lead to similar cognitive impairments.

Distorted perception experience in individuals with BDD led authors to speculate that they tend to process partial visual information rather than regulating classifications. Neuroimaging studies have supported this finding, indicating more hyperactivity in the left hemisphere associated with accurate analysis than the right hemisphere, which is responsible for overall evaluation (see e.g., Labuschagne et al., 2011). Such a mechanism can explain the desire of individuals with BDD for looking in the mirror with a greater focus on one aspect of their bodily appearance. Thus, the emotional impulsivity and distress mechanism in BDD individuals may cause them to interpret their mild physical deformities more negatively. The abnormal performance of the limbic system (e.g., amygdala) may also contribute to such interpretation.

Our study showed no significant difference between both groups in ToL test. Similarly, Veale, Sahakian, Owen, and Marks (1996) did not report any deficits in people with OCD in ToL test. However, when OCD patients committed errors, they spent more time providing alternative solutions or reviewing subsequent responses compared with the healthy group. In addition, using the Tower of Hanoi test, Schmidtke et al. (1998) reported good health and normal conditions of OCD patients in executive planning.

The possible explanation for this result is that OCD patients can hardly set aside the main goal and plan another goal for solving a different problem. When an OCD patient commits an error, he or she acts like ordinary people using the strategy of producing solutions, but they spend much more time investigating whether their attempts really lead to correct solutions or not. Individuals with OCD also spend more time solving a problem with ideality and obsession in their accuracy. Besides, OCD patients are certain that they are not committing errors by making much more efforts to review their responses (one of the components of executive performance refers to planning and decision‐making). The following reasons could explain the cognitive deficits in OCD individuals and their slow thinking; (a) they are easily disturbed by distracting inner and outer stimuli, (b) they conduct an extreme review and examine responses to ensure that there is no error, (c) it is difficult to set aside the main goal and to achieve other ones once an error occurs. In our study, individuals with BDD significantly spent much more time on tests.

In conclusion, executive functions (also called executive control or cognitive control) refer to a family of top–down mental processes needed when you have to concentrate due to ill‐advised and insufficient intuition (Miller & Cohen, 2001). The tasks measuring executive functions engage many cognitive resources, including the attention of individuals (Buss & Kerr‐German, 2019). Indeed, attention is the most important aspect of human cognition and is known as concentration and consciousness about a task (Shiri et al., 2018). Therefore, when the components of executive function are active and do not interact with one another as among people with BDD, the quality of attention decreases. While studies could examine the parameters that contribute to the process of cognitive rehabilitation of these individuals, further research is needed to investigate the consequences of cognitive dysfunction in patients with BDD.

There are some limitations in the current study. First, providing laboratory conditions in the school environment is bound by limitations. Second, as more people with BDD were excluded from the study due to comorbidity with psychotic disorders and substance abuse disorders, the generalizability of the findings was limited. Moreover, possible variables (e.g., intelligence, computer self‐efficacy, or technology‐related control source) were not examined in this study because they are likely to affect the results. Thus, it is suggested future research replicates this study in adult samples and use other measurement methods as well as other components of executive functions. Furthermore, further studies could also examine the role of moderator variables such as intelligence, gender, computer self‐efficacy, and technology‐based control source as inclusion and exclusion criteria in their design.

## CONFLICT OF INTEREST

The authors declare no conflict of interest.

## AUTHORS CONTRIBUTIONS

SR wrote the manuscript and conducted all statistical analyses, LK co‐wrote the manuscript and provided data, and MJKA co‐wrote the manuscript and supervised the research. All authors reviewed the final manuscript.

### PEER REVIEW

The peer review history for this article is available at https://publons.com/publon/10.1111/brb3.1710.

## Data Availability

The data that support the findings of this study are available from the corresponding author upon reasonable request.
